# NSAID-Based Coordination Compounds for Biomedical Applications: Recent Advances and Developments

**DOI:** 10.3390/ijms23052855

**Published:** 2022-03-05

**Authors:** Ariana C. F. Santos, Luís P. G. Monteiro, Adriana C. C. Gomes, Fátima Martel, Teresa M. Santos, Bárbara J. M. Leite Ferreira

**Affiliations:** 1 Department of Chemistry & CICECO, Aveiro Institute of Materials, University of Aveiro, Campus Universitário de Santiago, 3810-193 Aveiro, Portugal; arianasantos@ua.pt (A.C.F.S.); luispedromonteiro@ua.pt (L.P.G.M.); adrianagomes@ua.pt (A.C.C.G.); teresa@ua.pt (T.M.S.); 2 Instituto de Investigação e Inovação em Saúde (i3S), R. Alfredo Allen 208, 4200-135 Porto, Portugal; 3 Department of Biomedicine–Unit of Biochemistry, Faculty of Medicine of Porto, University of Porto, 4200-319 Porto, Portugal; fmartel@med.up.pt

**Keywords:** NSAID-based coordination compounds, first-row transition metals, anti-tumor activity, antimicrobial properties, antioxidant activity, interaction with biomolecules, DNA, RNA, proteins

## Abstract

After the serendipitous discovery of cisplatin, a platinum-based drug with chemotherapeutic effects, an incredible amount of research in the area of coordination chemistry has been produced. Other transition metal compounds were studied, and several new relevant metallodrugs have been synthetized in the past few years. This review is focused on coordination compounds with first-row transition metals, namely, copper, cobalt, nickel or manganese, or with zinc, which have potential or effective pharmacological properties. It is known that metal complexes, once bound to organic drugs, can enhance the drugs’ biological activities, such as anticancer, antimicrobial or anti-inflammatory ones. NSAIDs are a class of compounds with anti-inflammatory properties used to treat pain or fever. NSAIDs’ properties can be strongly improved when included in complexes using their compositional N and O donor atoms, which facilitate their coordination to metal ions. This review focuses on the research on this topic and on the promising or effective results that complexes of first-row transition metals and NSAIDs can exhibit.

## 1. Introduction

In the era of emerging drug resistance, mainly by bacteria, designing potent and successful novel therapeutic agents has become a major concern in the area of bioinorganic chemistry [1,2]. After the serendipitous discovery of the anticancer effects of cisplatin, a platinum-based drug (*cis*−[Pt(NH_3_)_2_Cl_2_]) which proved to have a strong chemotherapeutic effect, a tremendous amount of research on coordination chemistry-based drugs has been produced. Cisplatin can bind to the purine bases of DNA and is able to cause DNA damage that, in turn, can cause cancer cell apoptosis [3]. However, some adverse side effects, such as toxicity, allergy, gastrointestinal disorders and kidney problems, plus the lack of selectivity and acquired resistance to this compound [4,5,6], led investigators to explore more selective and less toxic substituents. During this endeavor, in the past few years a large number of new metal complexes have been synthetized. Specifically, some first-row transition metals have been preferred, namely, copper, cobalt, manganese, nickel and zinc, not only because they are easily available and cheaper, compared to platinum, but also for their less toxic nature and biocompatibility in living systems [7,8,9]. Additionally, transition metals can play a very important role in metallodrugs’ design due to their cations’ unique coordination environments, charge variation possibilities, Lewis acidic character or redox properties [8,10,11].

Regarding the above-mentioned first-row transition metals, some of their biological/bioinorganic implications deserve to be highlighted. Copper, as Cu(II), is a *d*^9^ metal cation structurally and catalytically involved in several biological processes by serving as a cofactor of many metalloproteins [12,13,14] and by promoting numerous enzymatic processes, such as cellular respiration or the biosynthesis of neurotransmitters [7,12,15,16]. In living organisms, copper exists predominantly in the Cu(II) oxidized form—that is, in the cupric form [12]. However, due to its redox ability, copper can also exist in a more reduced form, i.e., Cu(I). Therefore, it can either behave as an antioxidant or a pro-antioxidant species, and can either neutralize or induce the production of reactive oxygen species (ROS) [13,16,17]. Cobalt is another transition metal that is normally found as Co(II) (*d*^7^) or Co(III) (*d*^6^) forms, even though eventually it can exhibit a wide range of oxidation states from −1 to +4. Co(III) is mainly found in cobalamin (vitamin B_12_) [18]. Nevertheless, in order to have cobalt ions be biochemically active—i.e., involved in metabolic functions, such as fatty acid and amino acid metabolism [4,17,19] and regulating DNA, albeit indirectly [4,9,20,21]—cobalt needs to adopt Co(II) or Co(III) oxidation states. Manganese is also an essential element, and when as Mn(II), *d*^5^, it is associated with various physiological processes, such as development, reproduction and immune functions, energy metabolism and antioxidant defense [22]. Furthermore, it is involved in the synthesis and activation of several enzymes (e.g., transferases, hydrolases and isomerases) by acting as a cofactor [17,23]. In particular, in the central nervous system, the manganese ions act as cofactors for glutamine synthetase (GS) [24], and consequently an extreme exposure to this element is often linked to neurologic pathologies [2,24,25]. Manganese can exist in seven oxidation states (0, II–VII), but the biologically most important is Mn(II), which is inherently stable. In contrast, Mn(III) is unstable under acidic conditions, Mn(V) is unstable under all conditions and Mn(VII) is a strong oxidant species [26,27,28,29]. Nickel, with a *d*^8^ electronic configuration, Ni(II), is an essential element, although it still has, to a certain degree, few unclear biological functions [30]. It was originally found in the active center of urease [30,31,32], a non-mammalian enzyme that catalyzes the hydrolysis of urea, but with time, other nickel-dependent and nickel-containing enzymes were discovered [33]. Ni(II) ions are mainly found associated with nucleic acids in humans, since they coordinate with DNA’s nitrogen-containing bases [34]. It is also involved in proteins structurally and functionality [30]. Finally, zinc, another essential element, although not a transition metal by IUPAC definition [35], shows similar chemical properties to its periodic table neighbors, transition metals. As Zn(II), it possesses a 3*d*^10^ electronic configuration with all 3*d* orbitals fully filled. Therefore, even though Zn(II) complexes do not possess ligand field energy stabilization, they show wide coordination flexibility, especially with O donor atoms of amino acids or proteins [17]. Consequently, these characteristics (coordination numbers and structural variety) are critical in what concerns their catalytic roles in metalloenzymes, providing different interaction possibilities with substrates [36]. Zinc possesses a minor plasma pool and has rapid turnover. It is involved in several steps of cellular metabolism and is involved in respiration, immune functions, DNA synthesis and cell division [32,37,38].

## 2. History and Applications of Metallodrugs

The discovery of cisplatin in the 1960s triggered, firstly, the synthesis of new platinum compounds bearing biological activity, and then the preparation of complexes with other metals [39,40]. More recently, researchers have pursued complexes containing active drugs as ligands, since it has been proved that this approach could be a suitable strategy for developing new and more efficacious pharmacological compounds, together with less toxic effects when compared to the parent drugs [41,42,43,44,45]. Although some metal complexes received attention due to the anticancer activity of cisplatin and its derivatives, in many cases they possess other different and promising biological activities. Consequently, metallodrugs have been found to have innumerous applications, including anti-microbial, anti-inflammatory, anti-viral, anti-arthritic or anti-diabetic, and activities in relation to cardiovascular or gastrointestinal disorders as well [1,46,47,48,49].

**Figure 1:**
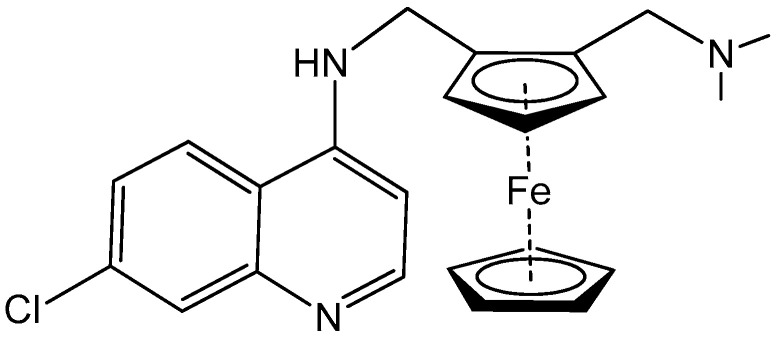
Ferrochloroquine’s chemical structure.

The first metallodrugs used in therapy were arsenic-based antimicrobial and antiparasitic agents. In particular, melarsoprol, an arsenic-based drug, is still used against trypanosomiasis [1], but other metal-based drugs with similar properties have emerged and are now commercially available [1,50]. One example is ferrochloroquine, an antimalarial agent, which is now undergoing phase II clinical trials. It is, more precisely, an organometallic compound (see Figure 1) [1,51].

Taking into account their possible biological activities, new coordination compounds with first row transition metals have been relentlessly investigated. Nowadays, particular attention is directed towards complexes with copper, cobalt, nickel, manganese or zinc, all of them possessing prominent biological effects [46,47,49,52,53]. 

One of the current focuses regarding these biometal complexes is their potential use as anti-inflammatory agents [7]. The synthesis of transition metal complexes with non-steroidal anti-inflammatory drugs (NSAIDs), used as ligands, started in 1978 with the preparation of an acetylsalicylic acid (aspirin) copper(II) coordination compound [53]. This study was an important breakthrough, since this complex showed a stronger anti-inflammatory response, and less ulcerogenicity and irritation to the digestive tract, than aspirin itself [54]. Since then, other biometal−NSAID complexes have been synthetized and studied in relation to their pharmacological effects. It is in this niche of research that this review is focused.

NSAIDs are a diverse class of compounds with anti-inflammatory properties used to treat pain or fever by inhibiting the two cyclooxygenase (COX) isoenzymes (known as COX-1 and COX-2) and also lipoxygenase (LOX) [17,19,55,56,57]. According to their chemical structures and selectivity, NSAIDs can be separated into different classes. Most of them are nonselective and so inhibit both COX-1 and COX-2. This is the case for (i) acetylated salicylates, (ii) non-acetylated salicylates, (iii) propionic acids, (iv) acetic acids, (v) enolic acids, (vi) anthranilic acids and (vii) naphthylalanine, in which the active agents are acetylsalicylic acid, diflunisial, naproxen or ibuprofen, diclofenac or indomethacin, meloxicam or piroxicam, tolfenamic acid or mefenamic acid and nabumetone, respectively [58]. However, there is a class of NSAIDs, called “coxibs,” whose members known for being selective for COX-2 inhibitors (e.g., celecoxib), and therefore, they have different side effect profiles in the treatment of inflammation [58].

### 2.1. Biometal−NSAID Complexes: A Few Coordination Topics

Divalent metal ions Cu(II), Co(II), Mn(II), Ni(II) and Zn(II), as mentioned above, can have essential redox and catalytic activities through structural modifications to the molecules (i.e., NSAIDs) they bind. NSAIDs, using N and O donor atoms, are able to easily coordinate to these metal ion centers, and the resulting coordination compounds can show enhanced biological activity compared to their parent NSAIDs [4,7,20,59,60,61]. Combining NSAIDs for treatment is a suitable strategy to modulate the potential effects of some available drugs. In particular, it might be an attractive approach in chemotherapeutics to circumvent multidrug resistance and inflammation-induced metastatic cancers [62,63]. Additionally, the synthesis of new drugs with synergistic biologically active ligands (i.e., the synthesis of metal−NSAID complexes) is an alternative option for modulating the therapeutic efficiency of the referred NSAIDs [63].

Cu(II) complexes with a variety of coordination numbers are common (4, 5 or 6), unlike Cu(I) complexes, which are mainly four coordinated [64]. Nevertheless, the preferred coordination environment of Cu(II) is square planar because of the Jahn–Teller distorting effect of the Cu(II) *d*^9^ electronic configuration [65]. Then, the main difference between Cu(II) and Cu(I) quadropoly coordinated compounds is the square-planar geometry of the former compounds against the tetrahedral of the latter. This particular aspect can be extremely important in the electron-transfer efficiency of some metallo-enzymes’ active sites. In most cases, they require distorted tetrahedral geometries, so that almost no energy is spent in structural rearrangements upon electron transfer. Paradigmatic examples are blue, or type 1, copper metalloproteins, which are responsible for carrying out electron transfer in a wide range of biological systems with variable enzymatic architectures [66,67].

Although cobalt complexes can contain cobalt with various oxidation states, as previously stated, Co(III) and Co(II) complexes are the most predominant ones in biological systems [68,69]. Co(III) coordination compounds usually have a low-spin *d*^6^ configuration, forming almost exclusively ix coordinate complexes with regular octahedral or distorted octahedral geometries [68]. Co(II) ions, often in high-spin *d*^7^ electronic configuration, can generally form four or six coordinate complexes with tetrahedral (distorted) and octahedral geometries [68], respectively, although ive coordinate complexes can also be found [69]. Normally, Co(II) ions easily interact with chelating N and O donor ligands [17,70,71].

Manganese(II) ions prefer to exist in ix coordinate complexes. However, these Mn(II) complexes are often unstable and easily interact with other molecules/ligands which can modify their coordination spheres, with consequences to their interaction modes with specific enzymes [72,73,74].

Complexes of nickel(II), in turn, typically adopt a variety of octahedral, square planar or tetrahedral geometries. However, rarer, ive coordinated compounds may also be formed [75,76].

As mentioned before, despite the lack of ligand field energy stabilization, zinc(II) complexes show coordination flexibility. Various structures of Zn(II) complexes have been observed, although coordination, mainly in tetrahedral geometries, is the most commonly found [77], as it represent the optimal and least strained structure among polyhedral zinc compounds [78].

The NSAIDs, mainly because of their characteristic carboxylic acid functional groups, which are in their anionic (deprotonated) forms at physiological pH, can be used as ligands, as they easily coordinate to metal ions, in a great versatility of coordination modes. Indeed, these drugs are able to coordinate as mono and bidentate modes, or even as bridges originating polynuclear metal complexes [57,79,80]. In Table 1, Table 2, Table 3, Table 4 and Table 5, some examples of these type of coordination modes are given. This review also summarizes the biological activities of some Cu(II), Co(II), Mn(II), Ni(II) and Zn(II) metal coordination compounds with some NSAIDs, which are organized into different groups. However, to the best of our knowledge, copper(II), cobalt(II), nickel(II), manganese(II) and zinc(II) metal complexes with the NSAID naphthylalanine, a non-natural analogue of phenylalanine involved in the inflammatory process [81], are not structurally characterized yet.

#### 2.1.1. Copper(II) Complexes of NSAIDs

Copper(II)−NSAID are the most numerous among the metal−NSAID complexes [82]. The structurally characterized and enumerated Cu(II)−NSAID complexes are mostly mononuclear, with carboxylate groups in bidentate chelating mode—e.g., [Cu(difl)_2_(py)_2_] [83], [Cu(nap)(tpy)Cl] [84] or [Cu(dicl)_2_(temed)] [85], although a monodentate chelating mode can be observed for [Cu(asa)(aroy)(H_2_O)_2_] [86], [Cu(Hmel)_2_(dmf)] [87], [Cu(tolf)_2_(py)_2_(MeOH)_2_] [88] and [Cu(cxb)_2_Cl_2_] [89] (see Table 1). With the exception of [Cu(Hmel)_2_(dmf)] [87] and [Cu(cxb)_2_Cl_2_] [89] complexes that are five and four coordinate with distorted square planar and square-pyramidal geometries, respectively, the structurally characterized examples listed in Table 1 are 6-coordinate, exhibiting distorted octahedral geometry.

#### 2.1.2. Cobalt(II) Complexes of NSAIDs

All the reported cobalt(II)−NSAID coordination compounds (Table 2) are mononuclear, with the NSAID carboxylate group being coordinated to the metal ion in a monodentate binding mode as in [Co(asa)(Haroy)(H_2_O)Cl] [86], [Co(difl)_2_(MeOH)_4_] [90], [Co(nap)_2_(py)_2_(H_2_O)_2_] [91], [Co(dicl)_2_(py)_2_(H_2_O)_2_] [19] and [Cu(cxb)_2_Cl_2_] [89] complexes, with the exception of [Co(Hmel)_2_(EtOH)_2_] [92] and [Co(tolf)_2_(bipyam)] [93] ones, where the carboxylate groups of meloxicam and tolfenamate ligands, respectively, are coordinated in a chelating bidentate mode. The majority of compounds are six coordinate with a distorted octahedral configuration.

#### 2.1.3. Nickel(II) Complexes of NSAIDs

In all the listed mononuclear complexes of Ni(II) ion (Table 3), the carboxylate coordination group is always in a monodentate binding mode, excepting in the [Ni(nap)_2_(phen)(H_2_O)] [94] complex. In this last case, the two deprotonated naproxen ligands are coordinated to nickel in two different binding modes: one naproxen ligand is bound to nickel in a bidentate chelating mode, and the other one is coordinated in a monodentate fashion. Again, with the exception of the [Ni(cxb)_2_Cl_2_] [89] complex, the reported examples were found to be six coordinate with a distorted octahedral geometry.

#### 2.1.4. Manganese(II) Complexes of NSAIDs

Not a lot of examples with manganese and the specific selected NSAIDs are described in the literature yet. However, for the enumerated cases (see Table 4), it is possible to see a diversity in coordination. The complexes are mononuclear, dinuclear or trinuclear. The structurally characterized mononuclear Mn(II)−NSAID complexes have the NSAIDs’ carboxylate groups bound to the metal ion in a monodentate mode, as in the [Mn(nap)_2_(py)_2_(H_2_O)_2_] [95] complex, or in a bidentate one, as in [Mn(tolf)_2_(phen)(H_2_O)] [96]. The nonlinear trinuclear Mn(II) complex is structurally diverse. The six diclofenac ligands are deprotonated and coordinated to the manganese atoms in three different modes: three of the six diclofenac ligands are in a bidentate binding mode and form µ_1,3_-bridges between two Mn atoms; two diclofenac ligands are in a tridentate binding mode and form µ_1,1_-bridges between two Mn atoms; and the sixth diclofenac ligand is monodentately bound to a terminal Mn atom through an oxygen atom. Additionally, the three Mn atoms are six coordinate and exhibit distorted octahedral geometries.

#### 2.1.5. Zinc(II) Complexes of NSAIDs

Similarly to Mn(II)−NSAID complexes, Zn(II)−NSAID complexes present a high diversity of nuclearity: we report in Table 5, mononuclear ([Zn(difl)_2_(bipy)] [62], [Zn(nap)_2_(N_3_)_2_]Na_2_ [80] and [Zn(Hmel)_2_(EtOH)_2_] [92]); binuclear (Zn_2_(dicl)_4_(nic)_2_ [97]); and trinuclear ([Zn_3_(tolf)_6_(CH_3_OH)_2_] [98]) cases. In the mononuclear complexes, the carboxylate groups are in either monodentate or chelating bidentate modes. For the binuclear case, one diclofenac molecule is monodently coordinated, while the other is bidently coordinated. Finally, for the centrosymmetric trinuclear complex, the six tolfenamato ligands behave as deprotonated ligands in the bidentate mode, forming six bidentate carboxylate bridges. The central Zn atom is six coordinate with a distorted octahedral geometry. The basal plane of the octahedron is formed by four coordinated carboxylate oxygen atoms of four different tolfenamato bridging ligands, while at the axial positions there are the carboxylate oxygen atoms of the remaining two tolfenamato bridging ligands.

**Table 1:** Examples of copper(II) complexes with different NSAIDs.

Chemical Formula	NSAID		Chemical Structure	Biological Activity	Ref.
	NSAID Ligand	NSAID Coordinating Group			
[Cu(asa)(aroy)(H_2_O)_2_] ^(a)^	Aspirin	Acetylated salicylate	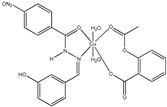 (proposed structure)	Weaker antimicrobial activity comparing to free aspirin.	[86]
[Cu(difl)_2_(py)_2_] ^(b)^	Diflunisal	Non-acetylated salicylate	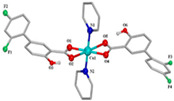	Moderate to strong DNA and albumin binding.	[83]
[Cu(nap)(tpy)Cl] ^(c)^	Naproxen	Propionic acid	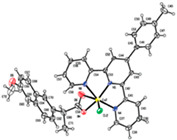	Hydrolytic DNA cleavage. Moderated cytotoxicity in a human breast cancer cell line (MCF-7).	[84]
[Cu(dicl)_2_(temed)] ^(d)^	Diclofenac	Acetate	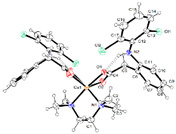	Significant reversible affinity for BSA, higher than the free NSAID sodium diclofenac.	[85]
[Cu(Hmel)_2_(dmf)] ^(e)^	Meloxicam	Enolic acid	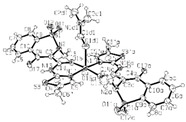	Possible beneficial effects as anticancer agent due to its anti-proliferative activity.	[87]
[Cu(tolf)_2_(py)_2_(MeOH)_2_] ^(f)^	Tolfenamic acid	Anthranilate	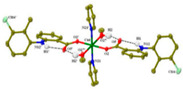	Tight binding affinity to BSA and HSA. Scavenging activity (against hydroxyl and superoxide radicals) stronger than free tolfenamic acid.	[88]
[Cu(cxb)_2_Cl_2_] ^(g)^	Celecoxib	Coxib	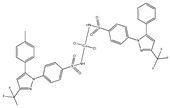 (proposed structure)	Inhibitory activity against Cyclooxygenase II.	[89]

^(a)^ asa = aspirin and aroy = aroylhydrazone *(m-hydroxylbenzaldehyde-4-nitrobenzoylhydrazone)*; ^(b)^ difl = diflunisal and py = pyridine; ^(c)^ nap = naproxen and tpy = substituted terpyridine; ^(d)^ dicl = deprotonated diclofenac and temed = *N*,*N*,*N*′,*N*′-tetramethylethylenediamine; ^(e)^ Hmel = protonated meloxicam and dmf = dimethylformamide; ^(f)^ tolf = tolfenamate and py = pyridine; ^(g)^ cxb = celecoxib.

**Table 2:** Examples of cobalt(II) complexes with different NSAIDs.

Chemical Formula	NSAID		Chemical Structure	Biological Activity	Ref.
	NSAID Ligand	NSAID Coordinating Group			
[Co(asa)(Haroy)(H_2_O)Cl] ^(a)^	Aspirin	Acetylated salicylate	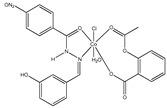 (proposed structure)	Weaker antimicrobial activity comparing to free aspirin.	[86]
[Co(difl)_2_(MeOH)_4_] ^(b)^	Diflunisal	Non-acetylated salicylate	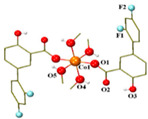	Radical scavenging ability and DNA and albumin binding.	[90]
[Co(nap)_2_(py)_2_(H_2_O)_2_] ^(c)^	Naproxen	Propionic acid	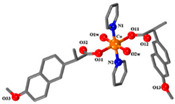	Good binding affinity to BSA and HSA and to DNA. High scavenging activity against hydroxyl and superoxide radicals.	[91]
[Co(dicl)_2_(py)_2_(H_2_O)_2_] ^(d)^	Diclofenac	Acetate	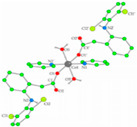	Antioxidant activity, DNA binding.	[19]
[Co(Hmel)_2_(EtOH)_2_] ^(e)^	Meloxicam	Enolic acid	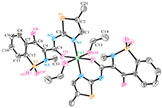	DNA biding and photocleavage of pUC57 plasmid DNA.	[92]
[Co(tolf)_2_(bipyam)] ^(f)^	Tolfenamic acid	Anthranilate	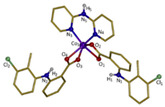	Good binding affinity to BSA and HSA and higher affinity to bind DNA comparing to free tolfenamic acid.	[93]
[Co(cxb)_2_Cl_2_] ^(g)^	Celecoxib	Coxib	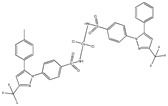 (proposed structure)	Inhibitory activity against cyclooxygenase II	[89]

^(a)^ asa = aspirin and Haroy = protonated aroylhydrazone *(m-hydroxylbenzaldehyde-4-nitrobenzoylhydrazone)*; ^(b)^ difl = diflunisal; ^(c)^ np = naproxen and py = pyridine; ^(d)^ dicl = deprotonated diclofenac and py = pyridine; ^(e)^ Hmel = protonated meloxicam; ^(f)^ tolf = tolfenamate and bipyam = 2,2′-bipyridylamine; ^(g)^ cxb = celecoxib.

**Table 3:** Examples of nickel(II) complexes with different NSAIDs.

Chemical Formula	NSAID		Chemical Structure	Biological Activity	Ref.
	NSAID Ligand	NSAID Coordinating Group			
[Ni(asa)(aroy)(H_2_O)_2_] ^(a)^	Aspirin	Acetylated salicylate	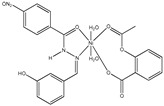 (proposed structure)	Weaker antimicrobial activity comparing to free aspirin.	[86]
[Ni(difl)_2_(MeOH)_4_] ^(b)^	Diflunisal	Non-acetylated salicylate	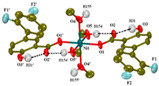	Albumin and DNA interaction, antioxidant activity.	[31]
[Ni(nap)_2_(phen)(H_2_O)] ^(c)^	Naproxen	Propionic acid	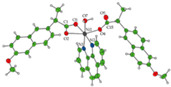	Significant affinity for BSA and HSA, DNA-binding and antioxidant activity.	[94]
[Ni(dicl)(Hdicl)(Hpko)_2_](dicl) CH_3_OH•0.6H_2_O ^(d)^	Diclofenac	Acetate	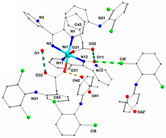	DNA and albumin binding.	[99]
[Ni(Hmel)_2_(H_2_O)_2_]•2H_2_O ^(e)^	Meloxicam	Enolic acid	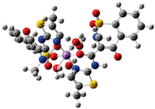 (theoretical structure)	Greater antibacterial activity than free meloxicam.	[100]
[Νi(tolf)_2_(bipy)(CH_3_OH)_2_] ^(f)^	Tolfenamic acid	Anthranilate	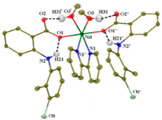	Significant affinity to bind BSA and HSA. Potent scavenging activity of hydroxyl and superoxide radicals. Better DNA binder comparing to free tolfenamic acid.	[101]
[Ni(cxb)_2_Cl_2_] ^(g)^	Celecoxib	Coxib	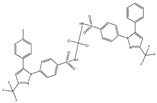 (proposed structure)	Inhibitory activity against cyclooxygenase II.	[89]

^(a)^ asa = aspirin and aroy = aroylhydrazone *(m-hydroxylbenzaldehyde-4-nitrobenzoylhydrazone)*; ^(b)^ difl = diflunisal; ^(c)^ np = naproxen and phen = 1,10-phenanthroline; ^(d)^ dicl = deprotonated diclofenac and Hpko = protonated 2,20-dipyridylketone oxime; ^(e)^ Hmel = protonated meloxicam; ^(f)^ tolf = tolfenamate and bipy = 2,20 -bipyridine; ^(g)^ cxb = celecoxib.

**Table 4:** Examples of manganese(II) complexes with different NSAIDs.

Chemical Formula	NSAID		Chemical Structure	Biological Activity	Ref.
	NSAID Ligand	NSAID Coordinating Group			
[{Mn(asa)(nic)}_2_(H_2_O)Cl]Cl•2H_2_O ^(a)^	Aspirin	Acetylated salicylate	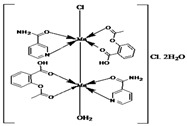 (proposed structure)	Comparing to standard (ascorbic acid) similar antioxidant activities were observed for the Mn(II) complex and both free drugs.	[102]
[Mn(nap)_2_(py)_2_(H_2_O)_2_] ^(b)^	Naproxen	Propionic acid	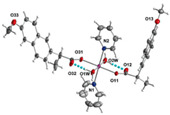	Selective scavenging activity of hydroxyl and superoxide radicals. Binds tighter to CT-DNA than the corresponding free NSAID and exhibits significant affinity to BSA and HSA.	[95]
[Mn_3_(dicl)_6_(phen)_2_(MeOH)] ^(c)^	Diclofenac	Acetate	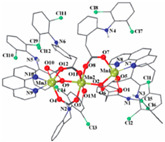	Higher binding affinities to BSA and HSA comparing to those of free sodium diclofenac. Significant ability to scavenge ABTS and hydroxyl radicals. Potent inhibitory activity of soybean lipoxygenase.	[103]
[Mn(Hmel)(Gly)(H_2_O)_2_]•5H_2_O ^(d)^	Meloxicam	Enolic acid	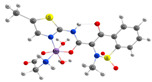 (DFT–optimized geometry)	No antifungal activity against *A. niger*, but antibacterial activities comparing to amoxycillin/clavulanic and cetaxime (antibacterial agents).	[104]
[Mn(tolf)_2_(phen)(H_2_O)] ^(e)^	Tolfenamic acid	Anthranilate	* 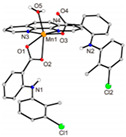 *	High scavenging activity against superoxide and hydroxyl radicals. It can also inhibit the activity of soybean lipoxygenase and shows tight binding affinity for BSA and HAS.	[96]

^(a)^ asa = aspirin and nic = nicotinamide; ^(b)^ np = naproxen and py = pyridine; ^(c)^ dicl = deprotonated diclofenac and phen = 1,10-phenanthroline; ^(d)^ Hmel = protonated meloxicam and gly = glycine; ^(e)^ tolf = tolfenamate and phen = 1,10-phenanthroline.

**Table 5:** Examples of zinc(II) complexes with different NSAIDs.

Chemical Formula	NSAID		Chemical Structure	Biological Activity	Ref.
	NSAID Ligand	NSAID Coordinating Group			
[Zn(asa)_2_] ^(a)^	Aspirin	Acetylated salicylate	No crystal structure has been published to the best of our knowledge.	After oral administration to rats it caused a decrease in blood glucose levels, and type-2 diabetes-induced damage in rat cardiac tissue was alleviated. This complex also showed a better post-ischemic myocardial dysfunction- preventing effect than free aspirin.	[105,106,107]
[Zn(difl)_2_(bipy)] ^(b)^	Diflunisal	Non-acetylated salicylate	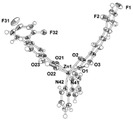	The complex is a more active radical scavenger and lipooxigenase inhibitor than free diflunisal. The complex also binds strongly to albumins.	[62]
[Zn(nap)_2_(N_3_)_2_]Na_2_^(c)^	Naproxen	Propionic acid	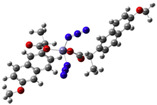	The complex shows antibacterial activity against Gram-positive (*S. aureus*) and Gram-negative (*E. coli*) bacterial strains.	[80]
[Zn_2_(dicl)_4_(nic)_2_] ^(d)^	Diclofenac	Acetate	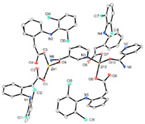	Improved solubility of the complex in comparision with free NSAID. The complex probably interacts with the grooves of the secondary structure of CT-DNA by electrostatic attraction.	[97]
[Zn(Hmel)_2_(EtOH)_2_] ^(e)^	Meloxicam	Enolic acid	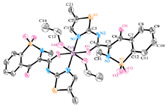	The complex may interact with DNA through an electrostatic mode and promoted the photo cleavage of a plasmid DNA.	[92]
[Zn_3_(tolf)_6_(CH_3_OH)_2_] ^(f)^	Tolfenamic acid	Anthranilate	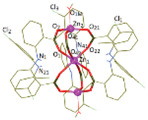	Good binding constants for BSA and HSA, suggesting a possible release from the serum albumin to the target cell.	[98]

^(a)^ asa = aspirin; ^(b)^ difl = diflunisal and bipy = 2,2′-bipyridine; ^(c)^ np = naproxen; ^(d)^ dicl = deprotonated diclofenac and nic = nicotinamide; ^(e)^ Hmel = protonated meloxicam; ^(f)^ tolf = tolfenamate.

As can be seen in Table 1, Table 2, Table 3, Table 4 and Table 5, whatever the metal ion in Cu(II), Co(II), Mn(II), Ni(II) and Zn(II) complexes, the ligand-drugs aspirin, diflunisal, naproxen, diclofenac and tolfenamic acid, are, in their deprotonated forms, coordinated by N and O donor atoms. All form six coordinated structures (regular or distorted octahedral geometries), except for the Cu(II)−meloxicam [87] complex, which has a square pyramidal geometry, as expected. With the ligand celecoxib, four coordinated structures have been proposed for complexes of copper, cobalt and nickel, as well [89]. However, despite a few exceptions, divalent metal−NSAID coordination compounds show a common tendency for octahedral geometries.

In general, all NSAID−metal complexes previously presented show increased biological activity compared to the parent drug, including antitumor, antimicrobial, antioxidant, and interactive activities. This enhancement of the biological activity of the metal complexes can be explained on the basis of Overton’s concept [108] and chelation theory [109,110]. According to the former concept, the cell membrane is selective and thus favors the crossing of lipid-soluble components. Upon chelation, in accordance with chelation theory, the polarity of the metal ion is reduced due to the overlapping with the ligand orbitals and partial sharing of the positive charge with donor groups. Chelation may also increase π−electron delocalization on the chelate ring, and therefore enhance the lipophilicity of the coordination compound, resulting in a better penetration into the cellular lipid membrane and consequent better biological activity [111].

## 3. Biological Effects of the Metal Complexes

### 3.1. Anti-Tumor Activity

Epidemiological studies found that there is a strong correlation between inflammation and cancer since there are phenotypical similarities between tumoral and inflammatory cells [112]. Cancer cells can induce tissue and DNA injuries through the secretion of inflammatory signals (cytokines, chemokines, etc.), which promote mutated cells to grow [112]. These cells, in turn, are able to produce more cytokines and recruit new inflammatory cells, creating an inflammatory environment that contributes to angiogenesis, migration and metastasis [112,113]. As metal−NSAID complexes have shown an enhanced affinity for DNA binding, they should be considered for tumor therapy [114]. Some metal−NSAID coordination compounds have shown to be selective for different cell lines, and so these types of “mixed compounds,” i.e., NSAIDs and metal ions, appear to be a very interesting step towards the selective killing of tumor cells [57,114]. According to literature, among the available NSAIDs−metal ion complexes, Cu(II)−NSAID complexes have the best anticancer effects, probably due to their well-known ability to reduce inflammatory processes [115,116]. Nevertheless, *Deb* and co-workers synthetized and characterized a Zn(II)–naproxen complex and a Zn(II)–mefenamate complex, which proved to have cytotoxic cell killing properties against a breast cancer cell line (MDA-MB-231) [117]. Of note, other metal-based compounds (besides the ones covered by this review) with anti-tumor potential, such as platinum−indomethacin and platinum−tolmetin complexes, have been proved to inhibit the growth of the L929 cancer cell line [118].

In 2012, *Sayen* and co-workers reported the first diclofenac-based coordination compound synthesized from a wholly aqueous medium. This compound exhibited cytotoxicity against human colon adenocarcinoma cell lines, and the Cu(II) salt and diclofenac have shown no cytotoxic activity *per si* [119]. More recently, a new Cu(II)−aspirin coordination compound was reported and revealed to be have multiple cellular targets (nucleus, mitochondrion and cyclooxygenase-2) [120]. This complex effectively induces mitochondrial dysfunction and promotes early apoptosis in ovarian cancer cells and also inhibits the expression of cyclooxygenase-2.

### 3.2. Antimicrobial Activity

Although NSAIDs are commonly used to treat pain, fever and inflammation, few reports suggest that NSAIDs also possess antimicrobial properties [121]. In particular, these drugs can be active against bacteria, viruses and fungi [122]. Their antimicrobial activity may be directly caused by membrane effects, metabolic alterations, DNA intercalation or adhesion suppression. However, indirectly, they can also serve as helper compounds, i.e., by having a synergistic effect, when co administered with other drugs, by inhibiting replication of the plasmids and eliminating them from cells, or stimulating cytokine production from the T-cells, or even potentiating the killing of the phagocytized microbes inside the macrophage [123]. Ibuprofen is one of the most widely used NSAIDs. Its antibacterial and antifungal activities were firstly described by Hersh and co-workers [124] and by Sanyal and co-workers [125], respectively. However, these antimicrobial effects are normally achieved with a high dose (higher than the therapeutic one) [125], which has been a large reason for the synthesis of new coordination compounds bearing this drug in their composition. For example, *Abu Ali* and co-workers synthetized a Zn(II)−ibuprofen metal complex that showed antibacterial activity against Gram-positive (*M. luteus*, *S. aureus* and *B. subtilis*) and Gram-negative bacteria (*E. coli*, *K. pneumonia* and *P. mirabilis*) [126]. Many other coordination compounds with NSAIDs and different metal ions also show antimicrobial activity. As examples, Table 2 lists a cobalt−naproxen metal complex that exhibited moderate activity against five Gram-positive bacteria, eight Gram-negative bacteria and three fungi. Additionally, *Lawal* and co-workers reported an example of a Co(II)-aspirin complex that showed a marked inhibitory effect against *B. subtilis* [127]. Moreover, a Ni(II)−meloxicam [100] complex was found to have stronger antimicrobial activity than free meloxicam (Table 3), and a Mn(II)–meloxicam complex also proved to have better antibacterial activity against *E. coli*, *Coliform*, *S. aureus*, *S. typhi*, *Citrobacter* and *Listeria* compared to the free NSAID (Table 4) ([104]. Additionally, *Ashouri* and co-workers synthetized novel Co(II) and Mn(II)−diclofenac coordination compounds that showed enhanced inhibitory activity in comparison with free diclofenac and metal salts [128]. It is noteworthy that several NSAIDs have antiviral activity, namely, aspirin, ibuprofen, naproxen, acetaminophen and lornoxicam, being able to potently inhibit the entry of *Zika* virus into the cells [129]. However, to the best of our knowledge, no studies evaluating the potential of NSAID-based metal complexes as antiviral agents have been done yet.

### 3.3. Antioxidant Activity

Reactive oxygen (ROS) and reactive nitrogen species (RNS) are normally formed during physiological and metabolic processes (e.g., mitochondrial respiration or inflammation) [130]. However, excessive production of these reactive species plays a critical role in the generation of oxidative stress. It is important to ensure a balance between the pro-oxidant and the antioxidant levels, in order to keep the biological equilibrium of the redox states in the cell [131,132,133]. Otherwise, the ROS/RNS may cause cellular, lipidic or DNA damage [130,134] that may result in many pathological conditions [135]. It is also known that lipid hydroperoxides and the oxygenated products of lipid degradation can contribute to cell proliferation, to signal transduction cascades and to differentiation and apoptosis [136]. Some NSAIDs have shown to be potent scavengers of ROS/RNS [134,137].

From the analysis of the Table 1, Table 2, Table 3, Table 4 and Table 5 (see above), we can see that most of the Cu(II)/Co(II)/Ni(II)/Mn(II)/Zn(II)−NSAID complexes have high antioxidant activity. Cu(II)/Ni(II)−tolfenamic acid complexes [88,101] proved to have strong scavenging activity compared to free tolfenamic acid, and the same behavior was observed for Co(II)/Ni(II)−diflunisal [31,90] and Co(II)/Ni(II)−naproxen [91,94] complexes. *Tarushi* and co-workers synthetized Zn(II)−diflunisal and Zn(II)−mefenamic acid complexes that proved to have an enhanced scavenging activity, compared with free drug, towards hydroxyl radicals [62,138]. *Dimiza* and co-workers also characterized Mn(II)−naproxen and Mn(II)−mefenamic acid complexes, which showed selective scavenging activity against hydroxyl and superoxide radicals [95]. Finally, some Zn(II)−tolfenamic acid complexes with low to moderate DPPH radical scavenging activity, but with a high scavenging activity against hydroxyl and superoxide radicals, have been also prepared [139].

## 4. Interactions with Biomolecules

### 4.1. Nucleic Acids

Most NSAIDs are found to have chemoprevention effects on different cell lines [140]. Their anticancer effects are proposed to be mainly exerted at the protein level and not at genomic level because NSAIDs have an anionic charge at physiological pH (which does not allow interaction with the polyanionic DNA backbone) [140]. In this context, the coordination of NSAIDs with biologically active metal ions, forming charge neutral complexes that may bind DNA, is a promising approach in order to improve the therapeutic action of NSAIDs or even to reduce their toxicity (such as the hepatotoxicity observed with aspirin) [141,142]. DNA binding can also be effective in antibiotic or antiviral therapeutic agents [92,143]. *Shahabadi* and co-workers showed that a Pt(II) complex containing ribavirin (an antiviral drug) interacts with DNA, probably via an intercalative mode, and that this complex has a higher affinity to DNA then ribavirin *per si* [144].

DNA is the primary macromolecule that is targeted by anticancer drugs. Small molecules can induce or suppress cellular interactions related to DNA, thereby changing its structure with inherent consequences for biological mechanisms, such as transcription, replication and repairing processes, which can consequently promote cell death [145,146]. In general, these interaction modes can be divided into two main types: covalent and non-covalent interactions [147]. A covalent bond can be formed when the complex has labile metal−ligand bonds. For example, in the case of cisplatin, the two chloride ligands are replaced by water molecules inside the cell. However, these water molecules are loosely bound to Pt, and a N atom of a nucleobase can displace them and allow the formation of a platinum−DNA covalent bond [94,148]. In a different approach, and in contrast with the DNA covalent interaction described above, metal complexes can interact with DNA in a reversible and non-covalent manner. This type of interaction offers several possibilities to contribute to the bonding, such as hydrogen bonding, π–π stacking and hydrophobic interactions [149]. This non-covalent bonding includes intercalation and groove binding [149,150]. Intercalation is anti-cooperative at adjacent sites, meaning that intercalators can only bind with alternative DNA base pairs. When an intercalator bonds to one DNA base pair, its two neighboring sites may continue unoccupied [151,152]. Groove binding corresponds to the association of the whole or a part of the complex with one of the grooves or with both of them (major and minor groove). This association is carried out by a combination of different parameters, such as electrostatic forces, van der Waals contacts, hydrophobic interactions and hydrogen bonding [153]. Once there are no free energy costs for this kind of binding, groove binders have bigger association constants than mere intercalators [149].

It is noteworthy that these alterations may result in improvements in the metal complexes’ biological activities, i.e., in the metallodrugs efficacy [125,154]. In particular, the interactions of metal−NSAID complexes with DNA, especially with calf-thymus DNA (CT-DNA), are considered of great importance when investigating the potential anticancer and/or anti-inflammatory effects of these coordination compounds, and for that reason, are the subject of many interaction studies [155].

#### Interactions of Cu(II)/Co(II)/Ni(II)/Mn(II)/Zn(II)−NSAID Complexes with DNA

By means of UV spectroscopy, it is possible to conclude that the bonding strength of the reported complexes (Table 1, Table 2, Table 3, Table 4 and Table 5, see above) to CT-DNA is strong, and in general, these metal−NSAID complexes bond more tightly to DNA than their parent drugs. For instance, the [Cu(nap)(tpy)Cl] [84], [Co(nap)_2_(py)_2_(H_2_O)_2_] [91], [Ni(nap)_2_(phen)(H_2_O)] [94] and [Mn(nap)_2_(py)_2_(H_2_O)_2_] [95] complexes have higher binding constants (Kb = 2.24 (±0.25) × 10^5^, 3.15 (±0.57) × 10^4^, 1.54 (±0.12) × 10^5^ and 2.29 (±0.13) × 10^5^ M^−1^, respectively) than the free NSAID naproxen (Kb = 2.67 (±0.22) × 10^4^). In addition, [Cu(nap)(tpy)Cl] [84], [Co(tolf)_2_(bipyam)] [93], [Ni(dicl)(Hdicl)(Hpko)_2_](dicl)CH_3_OH•0.6H_2_O [99], [Mn(nap)_2_(py)_2_(H_2_O)_2_] [95] and [Zn(Hmel)_2_(EtOH)_2_] [92] exhibit the highest K_b_ values among the listed Cu(II), Co(II), Ni(II), Mn(II) and Zn(II) complexes, respectively (see Table 6 below).

Through cyclic voltametric titration studies and viscosity measurements, it is possible to deduce how the complexes bind to DNA. Looking to the given examples in Table 1, Table 2, Table 3, Table 4 and Table 5, intercalation is the most common bonding mode to DNA. Three exceptions can be seen for [Cu(nap)(tpy)Cl] [84], [Zn_2_(dicl)_4_(nic)_2_] [97] and [Co(Hmel)_2_(EtOH)_2_] [92]. In the first two cases, the results demonstrated that the interactions between the complexes and DNA are due to groove binding events, and in the last case the spectroscopic and electrochemical results for Co(II)–meloxicam indicated that the complex can interact with DNA through an electrostatic mode.

Binding studies with the other nucleic acid, RNA, have been less frequent. Recently, this gap has been filled, due to the advantage that RNA offers much more extensive structural diversity than DNA [156]. Therefore, one may expect higher specificity of the compound’s interactions with RNA, through RNA-binding sites [157]. A recent study demonstrated that in the cisplatin treatment of *Saccharomyces cerevisiae*, the platinum complex accumulates 4 to 20-fold more times in cellular RNA than in genomic DNA [158]. With NSAIDs, *Huzaifa* and co-workers synthetized novel Cu(II) and Zn(II) coordination compounds, and mefenamic acid was used in their synthesis (although this ligand behaves as a counterion) [157]. These complexes demonstrated their preferential binding to t-RNA, when compared to CT-DNA, supporting the hypothesis that RNA interaction studies may be a new path to explore in the search for the synthesis of new and more efficacious metallodrugs. Regarding the possible antiviral activity of NSAIDs, the study of RNA binding affinity would entail greater importance, since most viruses are RNA viruses. In fact, there are indications that naproxen may bind to RNA groove of the influenza A virus [159], resulting in a novel antiviral drug. However, to the best of our knowledge, no studies to evaluating the interactions between metal−NSAID complexes and RNA viruses have been performed yet.

### 4.2. Proteins

For decades, metallodrugs research has been focused on DNA binding which, as described above, relies on the damage that metal complexes could inflict on DNA structure. However, it is now known that metallodrugs can exert their effects on other molecules, and proteins have appeared as some of their alternative targets [160]. Of all the molecules present in living organisms, proteins are the most abundant, and they are involved on almost every process. Kinase proteins may be good examples/targets, as their deregulation usually occurs in tumors, which can be crucial for the survival and progression of cancer cells [161]. There are already a few metal complexes that have been designed for the purpose of interacting with the ATP binding sites of kinase proteins and to act as their inhibitors [162,163]. It has been reported that some NSAIDs inhibit telomerase activity, an enzyme that plays a crucial role on a variety of cancers [164,165]. However, to the best of our knowledge, there are no reports of similar assays with metal−NSAID complexes. The same line of reasoning is applied for studies with G-quadruplex structures, which can be very interesting targets for metal complexes as well, since interactions between these structures and metal complexes may block telomerase activity, and consequently inhibit cancer cells to maintain telomere lengths [166,167]. However, no studies have been reported with metal−NSAID complexes, although a manganese coordination compound, a Mn(III)−porphyrin complex, has shown exceptional 10,000-fold selectivity for the telomeric region of duplex DNA [168].

Nevertheless, proteins in cancer cells are not the only target proteins. Host proteins can also act as “self” drug delivery systems. In this field, human serum albumin (HSA) is one of the most interesting systems [169]. HSA is the most abundant protein in blood plasma (60% of the total protein content), and because of its chemical characteristics, it can reversibly bind to drugs. This behavior, already known to occur with some NSAIDs, such as aspirin and ibuprofen, can enhance drugs’ biodistribution and/or bioavailability [160,170]. This issue, that is, drug–protein interactions, has been enlarged to include metallodrug−protein interactions. 

#### Interactions of Cu(II)/Co(II)/Ni(II)/Mn(II)/Zn(II)−NSAID Complexes with Serum Albumins

HSA and its structural homologue bovine serum albumin (BSA) have been the serum albumins used to study interactions with complexes, through quenching studies. From the Scatchard equation and graphs, the binding constants K have been calculated to determine the binding affinities of complexes to serum albumins. Various Cu(II)/Co(II)/Ni(II)/Mn(II)/Zn(II)−NSAID complexes have significant affinity for HSA (and BSA) proteins (see Table 1, Table 2, Table 3, Table 4 and Table 5 above). Among those examples, Cu(II)/Co(II)/Ni(II)/Zn(II)−diflunisal [31,62,83,90], Co(II)/Ni(II)/Mn(II)−naproxen [91,94,95], Cu(II)/Ni(II)/Mn(II)−diclofenac [85,99,103] and Cu(II)/Co(II)/Ni(II)/Zn(II)−tolfenamic acid [88,93,98,101] complexes can be highlighted due to high K values (see Table 7 below), revealing significant affinities for HSA, and tight but reversible binding to these albumin molecules.

It is noteworthy that all K values of the given examples are within an optimal range; i.e., in general they are higher than those of the free corresponding NSAIDs, allowing the binding of the complexes to serum albumins. However, these values are well below the association constant of one of the strongest known non-covalent bonds, the avidin–ligand interaction (K ≈ 10^15^ M^−1^), suggesting a possible release from the serum albumin to the target cells [82].

## 5. Conclusions

During the past 60 years, and after the success of platinum complexes for cancer treatment, metal-based drugs have been studied and nowadays some are commercially available. In particular, metal complexes containing NSAIDs are a group of compounds that have attracted much interest among the scientific community. More specifically, d-block metals and their cations, namely, copper, cobalt, nickel, manganese and zinc are by far the most exploited in NSAID-based metal complexes (metallodrugs).

In this review, the focus has been on the remarkable effects of these metallodrugs, including their wide ranges of biological activities (as anti-tumor, antimicrobial or antioxidant agents), and also on their ability to interact with nucleic acids. Since the pharmacologic effects of NSAIDs can be altered upon coordination to metal ions, it is possible to enhance the biological effects of the drugs and to decrease possible side effects, and eventually it may allow the interaction with new biomolecular targets. In this last case, the present review reports the potential of these coordination compounds as possible candidates for RNA targeting, since this last biomolecule not only plays important roles in cell and molecular biology (e.g., protein synthesis, messenger of genetic information, etc.), but also offers more extensive structural diversity than DNA that could be beneficial for RNA-binding metallodrug therapeutics. However, this review also considered protein targets (HSA, BSA), since binding to these proteins may be also of interest as targets or for the delivery of metallodrugs in chemotherapy. Moving toward a better understanding of these non-DNA molecular targets and their interactions with metallodrugs may be an advantageous path for the further optimization and consequent clinical development of metal−NSAID compounds.

## Figures and Tables

**Table 6:** DNA biding constants (K_b_) of Cu(II)/Co(II)/Ni(II)/Mn(II)/Zn(II)−NSAID complexes.

NSAIDs	K_b_ (M^−1^)	Cu(II)	K_b_ (M^−1^)	Co(II)	K_b_ (M^−1^)	Ni(II)	K_b_ (M^−1^)	Mn(II)/ Zn(II)	K_b_ (M^−1^)
Diflunisal (difl)	3.08 (±0.15) × 10^3^	Cu(II)-difl	7.36 (±0.11) × 10^4^	Co(II)-difl	2.26 (±0.12) × 10^5^	Ni(II)-difl	2.00 (±0.17) × 10^5^	-	-
Naproxen (nap)	2.67 (±0.22) × 10^4^	Cu(II)-nap	2.24 (±0.25) × 10^5^	Co(II)-nap	3.15 (±0.57) × 10^4^	Ni(II)-nap	1.54 (±0.12) × 10^5^	Mn(II)-nap	2.29 (±0.13) × 10^5^
Diclofenac (dicl)	3.16 (±0.14) × 10^4^	-	-	Co(II)-dicl	6.41 (±2.04) × 10^5^	Ni(II)-dicl	3.63 (±0.12) × 10^5^	-	-
Meloxicam (melox)	5.5 × 10^3^	-	-	Co(II)-melox	1.15 × 10^4^	-	-	Zn(II)-melox	5.34 × 10^4^
Tolfenamic acid (tolf)	5.00 (±0.10) × 10^4^	-	-	Co(II)-tolf	6.78 (±0.50) × 10^5^	Ni(II)-tolf	2.35 (±0.12) × 10^5^	-	-

**Table 7:** HSA biding constants (K) of Cu(II)/Co(II)/Ni(II)/Mn(II)/Zn(II)−NSAID complexes.

NSAIDs	K (M^−1^)	Cu(II)	K (M^−1^)	Co(II)	K (M^−1^)	Ni(II)	K (M^−1^)	Mn(II)/ Zn(II)	K (M^−1^)
Diflunisal (difl)	1.22 (±0.07) × 10^5^	Cu(II)-difl	7.36 (±0.11) × 10^4^	Co(II)-difl	2.26 (±0.12) × 10^5^	Ni(II)-difl_2_	1.41 (±0.08) × 10^5^	Zn(II)-dilf_2_	9.94 (±0.35) × 10^5^
Naproxen (nap)	5.35 × 10^3^	-	-	Co(II)-nap	3.15 (±0.57) × 10^4^	Ni(II)-nap_2_	2.73 (±0.25) × 10^4^	Mn(II)-nap_2_	6.50 (±0.30) × 10^4^
Diclofenac (dicl)	3.55 × 10^3^	Cu(II)-dicl	2.23 (±0.09) × 10^3^	Co(II)-dicl	6.41 (±2.04) × 10^5^	Ni(II)-dicl	2.54 (±0.27) × 10^4^	Mn(II)-dicl	1.86 (7) × 10^5^
Tolfenamic acid (tolf)	3.12 (±0.25) × 10^5^	Cu(II)-tolf	4.16 (±0.24) × 10^5^	Co(II)-tolf	6.78 (±0.50) × 10^5^	Ni(II)-tolf_2_	2.23 (±0.11) × 10^5^	Mn(II)-tolf_2_	3.56 (±0.13) × 10^5^
								Zn(II)-tolf	4.12 × 10^5^
